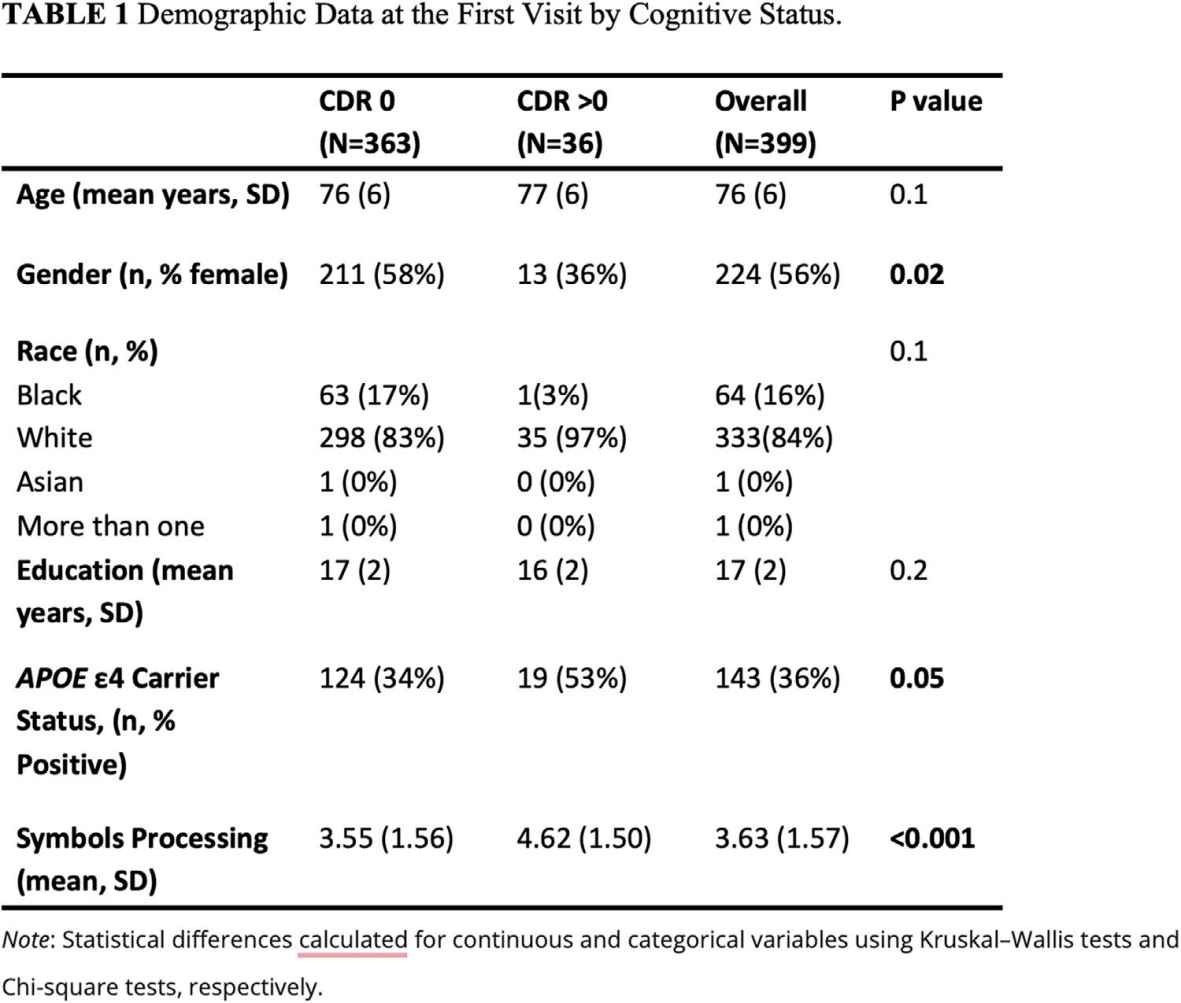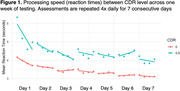# Examining Practice Effects at Multiple Retest Intervals in Older Adults at Risk for Alzheimer's Disease

**DOI:** 10.1002/alz.086319

**Published:** 2025-01-03

**Authors:** Hannah M Wilks, Matthew S. Welhaf, Andrew J. Aschenbrenner, Carlos Cruchaga, John C. Morris, Jason J. Hassenstab

**Affiliations:** ^1^ Washington University in St. Louis, St. Louis, MO USA; ^2^ Knight Alzheimer Disease Research Center, Saint Louis, MO USA; ^3^ Knight Alzheimer Disease Research Center, St. Louis, MO USA; ^4^ Washington University School of Medicine in St. Louis, St. Louis, MO USA; ^5^ Washington University in St. Louis, School of Medicine, St. Louis, MO USA; ^6^ NeuroGenomics and Informatics Center, Washington University School of Medicine, St Louis, MO USA

## Abstract

**Background:**

Practice effects (PEs) on cognitive tests are improvements in performance from repeated testing. We and others have shown that reductions in PEs on standard tests administered annually are associated with neurodegenerative disease. Using mobile technology, cognition can be assessed at much higher frequencies than standard in‐clinic assessments. These high‐frequency assessments offer a unique opportunity to measure PEs at multiple timescales, including retest intervals across months, weeks, days, within‐day, and even trial‐to‐trial. It is unclear if PEs observed across these different retest intervals are equally sensitive to clinical status and genetic risk of Alzheimer’s disease (AD).

**Method:**

We examined timescales of PEs using a very brief, smartphone‐based processing speed test in 399 well‐characterized older adults (Table 1). Cognition was assessed four times per day over a week every six months for up to 2.5 years. PEs from the task were modeled at multiple timescales, including biannually, weekly, daily, and at the trial level. Additional mixed effects models compared PEs across clinical status and genetic risk for AD.

**Result:**

Across all 399 participants, there were significant practice effects at all timescales, with an average improvement in speed of 0.10s over a 6‐month retest interval, 0.02s within each day of testing, and 0.005s each individual testing session. Performance improved throughout each week, and the most performance gains occurred on the first day of testing. Comparing clinical status using the Clinical Dementia Rating (CDR®) revealed that CDR>0s had significantly slower overall processing speed (Cohen's d = 0.9) than CDR0s, however, CDR>0s displayed much more performance gains than CDR0s on their first three days of testing (Figure 1; d = 1.4). Across biannual visits, CDR>0s lost more performance gains than CDR 0s, indicating less retention over time. Comparing APOE e4 carriers and e4 noncarriers revealed similar patterns. Carriers of the e4 allele were slower than noncarriers (d = 0.3) and lost more performance gains across biannual visits.

**Conclusion:**

Magnitudes of PEs across multiple retest intervals are sensitive indicators of clinical status and genetic risk for AD. PEs observed at the daily level appear to be the most sensitive to clinical status and genetic risk for AD.